# German Cardiac Arrest Registry: rationale and design of G-CAR

**DOI:** 10.1007/s00392-022-02044-9

**Published:** 2022-06-21

**Authors:** Janine Pöss, Christoph Sinning, Isabelle Schreiner, Christian Apfelbacher, Karl-Philipp Drewitz, Nadine Hösler, Steffen Schneider, Burkert Pieske, Bernd W. Böttiger, Sebastian Ewen, Harm Wienbergen, Malte Kelm, Daniel Bock, Tobias Graf, Christoph Adler, Jochen Dutzmann, Wulf Knie, Martin Orban, Uwe Zeymer, Guido Michels, Holger Thiele, the G-CAR Investigators

**Affiliations:** 1 Department of Internal Medicine/Cardiology, Heart Center Leipzig at University of Leipzig and Leipzig Heart Institute, Strümpellstr. 39, 04289 Leipzig, Germany; 2 University Heart and Vascular Center Hamburg, Hamburg, Germany; 3 Medical Faculty, Otto Von Guericke University Magdeburg, Magdeburg, Germany; 4 Leipzig Heart Science, Leipzig, Germany; 5 Institut Für Herzinfarktforschung, Ludwigshafen am Rhein, Germany; 6 Charité University Medicine, Campus Virchow Klinikum and German Heart Center and Berlin Brandenburger Center for Regenerative Therapies (BCRT) of the Berlin Institute of Health (BIH), Berlin, Germany; 7 Faculty of Medicine and University Hospital Cologne, Cologne, Germany; 8 University Hospital Saarland, Homburg/Saar, Germany; 9 Klinikum Links Der Weser, Bremen, Germany; 10 University Hospital Düsseldorf, Düsseldorf, Germany; 11 Klinikum Frankfurt Höchst GmbH, Frankfurt am Main, Germany; 12 University Heart Center Lübeck, Lübeck, Germany; 13 University Hospital Halle (Saale), Halle, Germany; 14 Charité University Medicine, Campus Benjamin Franklin, Berlin, Germany; 15 Klinikum der Universität München and DZHK (German Center for Cardiovascular Research), Partner Site Munich Heart Alliance, Munich, Germany; 16 St.-Antonius-Hospital gGmbH, Eschweiler, Germany

**Keywords:** Cardiopulmonary resuscitation (CPR), Out-of-hospital cardiac arrest (OHCA), Registry, Extracorporeal cardiopulmonary resuscitation (eCPR), Cardiac arrest centre (CAC), Post-resuscitation care

## Abstract

**Background:**

In Germany, 70,000–100,000 persons per year suffer from out-of-hospital cardiac arrest (OHCA). Despite medical progress, survival rates with good neurological outcome remain low. For many important clinical issues, no or only insufficient evidence from randomised trials is available. Therefore, a systemic and standardised acquisition of the treatment course and of the outcome of OHCA patients is warranted.

**Study design:**

The German Cardiac Arrest Registry (G-CAR) is an observational, prospective, multicentre registry. It will determine the characteristics, initial treatment strategies, invasive procedures, revascularisation therapies and the use of mechanical circulatory support devices with a focus on extracorporeal cardiopulmonary resuscitation. A special feature is the prospective 12-month follow-up evaluating mortality, neurological outcomes and several patient-reported outcomes in the psychosocial domain (health-related quality of life, cognitive impairment, depression/anxiety, post-traumatic stress disorder and social reintegration). In a pilot phase of 24 months, 15 centres will include approximately 400 consecutive OHCA patients ≥ 18 years. Parallel to and after the pilot phase, scaling up of G-CAR to a national level is envisaged.

**Conclusion:**

G-CAR is the first national registry including a long-term follow-up for adult OHCA patients. Primary aim is a better understanding of the determinants of acute and long-term outcomes with the perspective of an optimised treatment.

**Trial registry:**

NCT05142124.

**Graphical abstract:**

German Cardiac Arrest Registry (G-CAR)

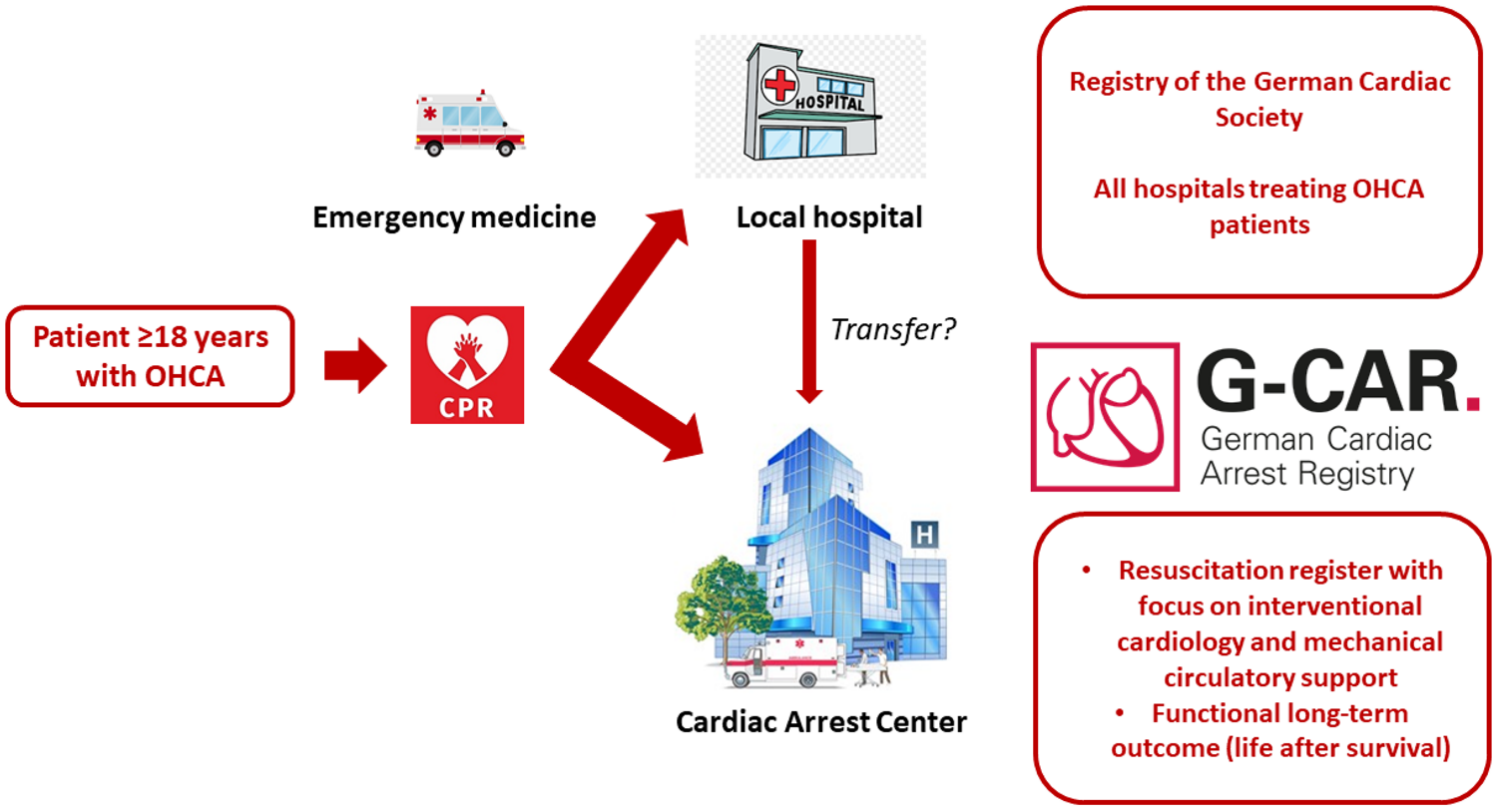

## Introduction

In Germany, 70,000–100,000 patients suffer from OHCA every year [1]. Over the last years, a trend towards a decrease in mortality of OHCA patients was observed [2, 3]. However, survival rates are still very low with 2–10% [4–6]. Therefore, optimisation of medical care with the aim to improve clinical outcomes of OHCA patients is crucial. For many clinical questions, no adequate evidence from randomised trials is available. Besides the conduction of randomised trials, a systematic and standardised recording of the pre-clinical, clinical and post-clinical treatment course and of the clinical outcomes of OHCA patients in a “real-world setting” is a prerequisite for an improvement of patient care. In over 60% of OHCA cases, a cardiac aetiology can be identified [7]. Therefore, cardiologists have an important role in the care of OHCA patients together with intensive care and emergency medicine physicians [8, 9]. Cardiac arrest centres play an important role for patient management and outcome and recently a structured certification has been introduced with the aim to further improve outcome [10, 11]. A systematic documentation of patients is required for the certification of cardiac arrest centres [12] and is recommended in the revised S3 guidelines on infarct-related cardiogenic shock [13]. Existing registries such as the German Resuscitation Registry of the German Society of Anaesthesiology and Intensive Care Medicine or smaller registries mainly focus on pre-clinical and clinical data [14]. A prospective registry collecting pre-clinical, clinical, post-clinical variables and treatments as well as long-term outcomes of OHCA patients is still missing. Patient-reported outcomes such as health-related quality of life, psychopathologic symptoms (i.e. cognitive impairment, depression, anxiety or post-traumatic stress disorder) and return to normal life increasingly get into the scientific focus since they provide important information outcomes from the patients’ perspective [15]. G-CAR will be the first multicentre German registry covering all the above-mentioned parameters. Aim of G-CAR is to achieve a better understanding of acute and long-term consequences of OHCA, to provide a nationwide quality assurance and to identify and optimise processes during treatment and follow-up of OHCA patients.

## Study design

### Study objectives

**Fig. 1:**
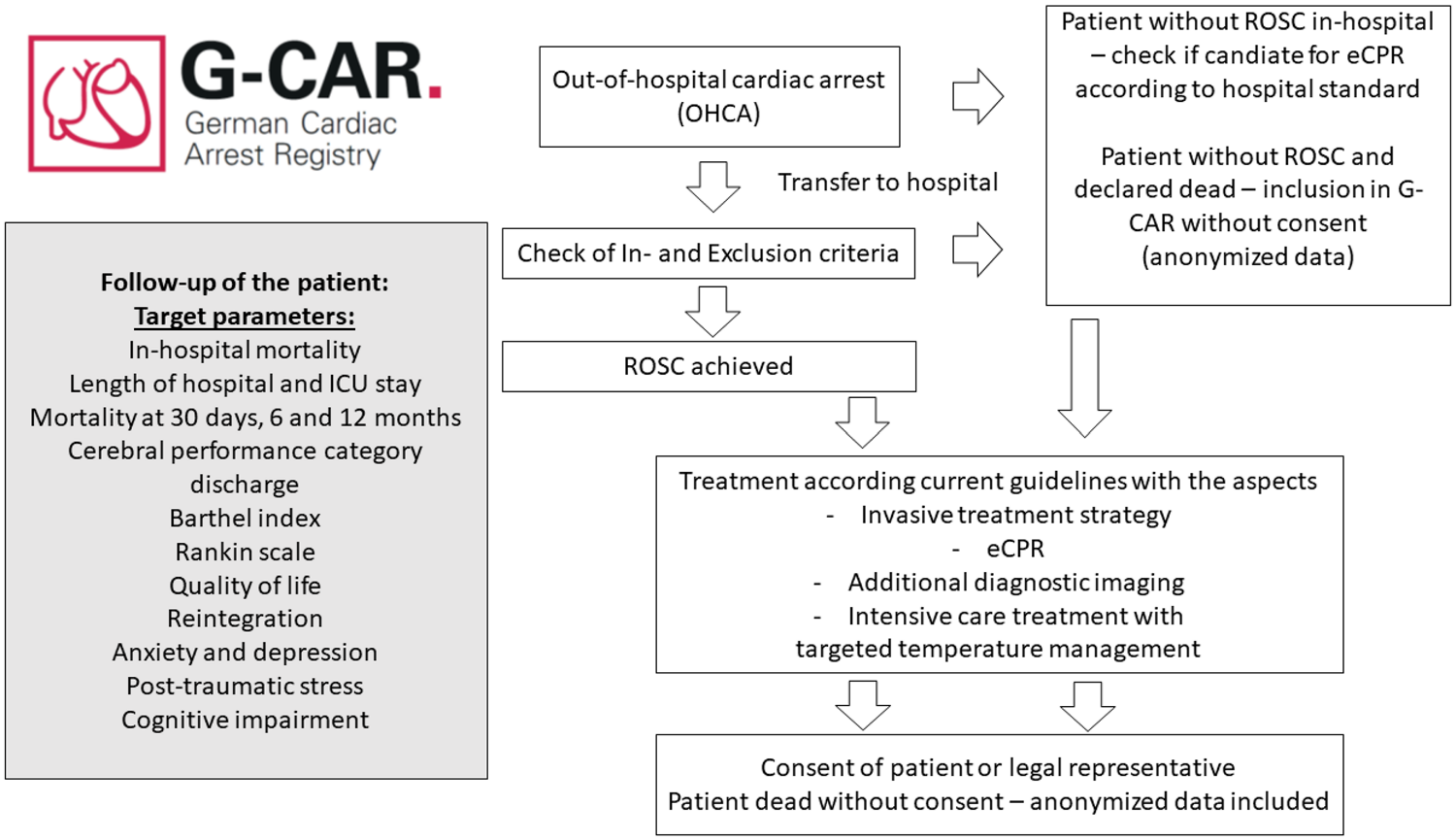
G-CAR—registry flow chart (*OHCA* out-of-hospital cardiac arrest, *ROSC* return of spontaneous circulation)

G-CAR is a prospective, national, multicentre registry in patients with OHCA, Fig. 1.

It will cover three phases of treatment:

1. Pre-hospital phase
2. Hospital phase divided into (a) acute phase after cardiopulmonary resuscitation (emergency department or catheterisation laboratory), (b) invasive and interventional cardiology (intervention phase), (c) treatment on the intensive care unit (ICU) (critical care phase)
3. Post-hospital care and outcomes (e.g. quality of life, rehabilitation phase).

Furthermore, data on new treatment options, such as extracorporeal cardiopulmonary resuscitation (eCPR) will be investigated. Therefore, the G-CAR registry aims to build a link to the field of patient care research. To achieve this, a cooperation with the working groups of the German Cardiac Society related to clinical acute and emergency medicine/intensive care medicine was initiated. Due to the important scientific need of such a registry, the German Cardiac Society (DGK) has agreed to endorse the project. Therefore, the registry will be under the aegis of the DGK. G-CAR is registered at www.clinicaltrials.gov (NCT05142124) and can be viewed on the website *cardiacarrestregistry.de*.

### Target parameters

In-hospital mortality, length of ICU and hospital stay as well as neurological outcomes will be recorded. Follow-up assessment will be performed at 30 days, 6 months and 12 months after OHCA and include data on clinical and neurological outcomes as well as on patient-reported outcomes. Data acquisition during hospitalisation and follow-up is displayed in Table 1.

**Table 1:** Diagram of data acquisition

	Baseline/hospital stay	Discharge	Follow-up 30 days	Follow-up 6 months	Follow-up 12 months
Informed consent	x				
Baseline characteristics	x				
Comorbidities	x				
CPR details/prehospital treatment	x				
Data on intrahospital treatment	x				
Rehospitalisation			x^b^		
Mortality	x	x	x^b^	x^b^	x^b^
CPC	x^a^	x		x^b^	x^b^
Modified Rankin scale	x^a^	x		x^b^	x^b^
Barthel index		x		x^b/c^	x ^b/c^
T-MoCA			x^b^	x^b^	x^b^
Quality of life—EQ-5D				x^c^	x^c^
Anxiety/depression—HADS				x^c^	x^c^
Social reintegration—RNLI				x^c^	x^c^
Post-traumatic stress—PTSS-14				x^c^	x^c^

*CPR* cardiopulmonary resuscitation, *CPC* cerebral performance category, *EQ-5D* EuroQol-5D, *PTSS-14* Post-Traumatic Stress Syndrome 14-Questions Inventory, *HADS* Hospital Anxiety and Depression Scale, *RNLI* Reintegration to Normal Living Index, *T-MoCA* Montreal Cognitive Assessment

^a^Before OHCA

^b^Telephone interview

^c^Questionnaire

Full list of target parameters:

- In-hospital mortality
- Length of ICU stay
- Length of hospital stay
- Cerebral performance category (CPC) at discharge
- Rankin scale at discharge, 6 months and 12 months
- Activities of daily living assessed by Barthel index at discharge, 6 months and 12 months
- Mortality at 30 days, 6 months and 12 months
- Health-related quality of life assessed by EuroQol-5D (EQ-5D) at 6 months and 12 months
- Post-traumatic stress disorder assessed by Post-Traumatic Stress Syndrome 14-Questions Inventory (PTSS-14) at 6 months and 12 months
- Social Reintegration assessed by Reintegration to Normal Living Index (RNLI) at 6 months and 12 months
- Anxiety and Depression assessed by Hospital Anxiety and Depression Scale (HADS) at 6 months and 12 months
- Cognitive Impairment assessed by Montreal Cognitive Assessment (T-MoCA)

### Patient population

The study population will consist of approximately 400 OHCA patients enrolled at 15 centres in Germany. Recruitment has commenced in 08/2021 and as of 04/2022 47 patients have been enrolled.

#### Inclusion criteria

- Out-of-hospital cardiac arrest
- Age ≥ 18 years

#### Exclusion criteria

- Rejection of register participation by patient or legal representative

#### Informed consent

Due to the severity of the disease, most of the patients will not be able to consent at hospital admission. All patients who become able to consent during hospital stay will be asked for consent as soon as possible. If patients do not regain their consciousness including their capacity to consent during hospitalisation, a legal representative will be asked for consent. Data of patients who die before they regain their capacity to consent and before a legal representative was appointed will be anonymised and included in the registry. This informed consent process has been validated and approved by the ethical committees in all recruiting study centres.

### Data and statistical analysis

Data collection is implemented by means of an electronic case report form (CRF) EBogen©, the electronic data capture (EDC) system of the Institut für Herzinfarktforschung (IHF). When entering data by the participating centres into the study database, the data is automatically checked for plausibility using a so-called online edit check. All requirements of the General Data Protection Regulation are met. In particular, the addresses of the patients for performing the follow-up assessments are stored separately from the clinical data.

Data will be analysed by descriptive statistics. Mortality during follow-up will be visualised using Kaplan–Meier curves. Regression models will be fitted to identify patient characteristics that are associated with the defined outcome parameters. The effects of the independent parameters will be estimated (odds ratio, hazard ratio) and given with 95% confidence intervals.

Due to the exploratory character of the project, statistical evaluations are possible at any time. Reports and publications are planned after the end of the three defined follow-up times (30 days, 6 and 12 months). Benchmarking reports will be made available to participating centres on a regular basis while the registry is ongoing. The report is created individually for each participating centre and compares the centre's data with the data from all other centres. This benchmarking system offers the participating centres the possibility of internal and external quality control.

### Study organisation

G-CAR follows a standard study organisation. G-CAR will be overseen by a steering committee chaired by Janine Pöss, MD and seven additional members. The steering committee is responsible for the scientific content of the protocol, oversees the trial operations, and will perform the preparation of the primary manuscript and other publications arising from G-CAR. All trial-related processes will follow the standard operating procedures of the IHF which acts as clinical research organisation and the Leipzig Heart Science (LHS) which takes on sponsor tasks. The IHF provides biometry, electronic case report form (eCRF) and data management whereas the LHS is responsible for study coordination, and also data management.

### Sources of funding

The G-CAR project is an investigator-initiated registry supported mainly by the German Cardiac Society (DGK)—Zentrum für kardiologische Versorgungsforschung (DGK-ZfKVF), the German Heart Research Foundation and the Dr. Rolf M. Schwiete Stiftung. The LHI and the individual centres provide additional funding. The authors are solely responsible for the design and conduct of this study, all study analyses, the drafting and editing of the paper, and its final contents. The above-mentioned foundations and organisations have no influence on the study design, data collection, data analysis, and final drafting of this manuscript.

### Treatment of patients and follow-up assessments

Patients will be treated according to the current standard of care. The decision about whether or not to perform a coronary angiography will be taken according to the international guidelines: patients with an ST-elevation myocardial infarction (STEMI) will undergo emergent coronary angiography. In other patients, the decision will be taken on an individual basis by the treating interdisciplinary team of medical specialists. Data regarding performance and timing of coronary angiography/PCI, access site, location of the culprit lesion (if present), number of diseased vessels and thrombolysis in myocardial infarction (TIMI) flow will be assessed. The indication regarding implementation of mechanical support (extracorporeal life support; ECLS) or eCPR will be made by the treating multidisciplinary team. If eCPR is performed, many details such cannulation technique, cannula size, left ventricular unloading, timing of implantation, duration of mechanical assist and complications will be assessed. Temperature management will be performed according to the local protocols. Neurological prognostication should be performed in cooperation with a specialised neurologist by a multimodal approach (measurement of neuron-specific enolase (NSE), cerebral imaging (computer tomography (CT) or magnetic resonance imaging (MRI)), electroencephalography (EEG), neurophysiological examinations with measurement of somatosensory-evoked potentials and neurological clinical exam) according to international and to local guidelines and should be done at earliest after 72 h [9]. During hospital stay, the results of different clinical and laboratory-chemical exams as well as instrumental procedures will be recorded. Furthermore, clinical and neurological outcomes will be assessed.

After 30 days, 6 and 12 months, the outcome of the patients will be assessed—either via personal interview if the patient is still hospitalised or via structured telephone interview by qualified staff. During the first phone calls, the investigators prepare the patients for the questionnaires which will be sent by regular mail during the next follow-ups and explain them the importance of the questionnaires for the study. After 6 and 12 months, patient-reported outcomes such as health-related quality of life and psychopathological symptoms (cognitive impairment, affective disorders, post-traumatic stress disorder) will be assessed by standardised questionnaires and telephone interview. If questionnaires are not sent back after 4 weeks, patients will be contacted by phone and mail. If patients cannot be reached, the respective registration office will be contacted in order to assess the survival state of the patients. A detailed description of the target parameters is provided above. In parallel to the pilot phase of 1 year, a national extension of G-CAR is planned.

## Discussion

Since G-CAR covers the prehospital, hospital and post-discharge phase, its data will shed some light on many unsolved or insufficiently solved issues regarding the treatment of OHCA patients. The most important clinical questions are described in more detail.

### Pre-hospital phase

Over the last 3 decades, the presence of shockable rhythms (ventricular tachycardia or fibrillation) declined, whereas non-shockable rhythms such as pulseless electric activity (PEA) and asystole have been more often reported [6, 16]. The main reasons for this development are the optimisation of coronary artery disease treatment and the ageing population resulting in more patients with advanced cardiovascular disease and with extensive extra-cardiac comorbidities. This patient cohort often suffers from sudden cardiac arrest due to acute respiratory or metabolic problems resulting in a non-shockable rhythm [6]. Incidence rates as well as predisposing conditions of OHCA are varying and differ according to region, age, ethnicity, race and sex. However, this disease entity still represents a major public health problem all over the world. Over the last years, resuscitation rates and, as a consequence, the number of patients initially surviving the acute event has increased in some countries. However, the majority of patients will still have a very high mortality due to post-cardiac arrest syndrome with post-cardiac arrest brain injury, myocardial dysfunction, and systemic ischaemia/reperfusion injury to various organ systems [6, 9, 17]. Consequently, a population-wide approach will be required to decrease the burden of OHCA [6, 16, 17].

### Hospital phase

#### Acute phase after cardiopulmonary resuscitation

Especially at the interface between prehospital and clinical care, a major challenge is to continue patient treatment without loss of time and at a high level. This includes the stabilisation of vital functions and the implementation of a suitable diagnostic and therapeutic strategy. In order to map the optimal quality of post-cardiac arrest shock room management, the most important parameters should be recorded (e.g. clinical status and ECG after return of spontaneous circulation (ROSC), imaging at admission of the patient).

#### Timing of coronary angiography

Acute coronary syndrome (ACS) is an important diagnosis in patients with OHCA. Current studies report its presence in 59–71% [9]. Early revascularisation by PCI is the guideline-based approach in patients with STEMI [18]. However, most OHCA patients show diffuse, non-specific ECG changes after ROSC. Furthermore, the increasing number of patients with non-shockable rhythms further complicates the decision regarding the indication and timing of coronary angiography. The Angiography After Out-of-Hospital Cardiac Arrest Without ST-Segment Elevation (TOMAHAWK) [19] and the Coronary Angiography after Cardiac Arrest (COACT) trials [20] are two randomised multicentre trials showing both no significant differences in clinical outcome among patients with OHCA between immediate and delayed coronary angiography [19, 20]. The COACT trial enrolled only patients with a shockable arrest rhythm, the TOMAHAWK trial recruited patients with both shockable and non-shockable rhythms. G-CAR will add information of current real-world practice and its association with clinical outcome and might identify predictors for the presence of an underlying coronary cause in OHCA.

#### Neurological prognostication

Neurological prognostication of OHCA patients is a clinical challenge and has far-reaching consequences for the further therapeutic management (e.g. therapy escalation or limitation). G-CAR will help to identify predictors for the neurological outcome of OHCA patients and might be the basis for the development of a prognostic score. In patients treated with ECLS, the NSE is not sufficiently evaluated for prognostication. Circulatory support devices and also conventional CPR may cause haemolysis, which might lead to an increase in NSE and thus reduce its prognostic power in these patients [21, 22]. It remains unclear whether the time point, the maximum value or the dynamic of NSE is/are of prognostic relevance [21–23]. Furthermore, the benefit of targeted temperature management during ELCS treatment is controversially debated [24–26]. G-CAR might help to answer these questions.

#### Extracorporeal cardiopulmonary resuscitation (eCPR)

Over the last years, the use of eCPR in the setting of OHCA has increased. Implantation of a veno-arterial extracorporeal membrane oxygenator (ECMO; also called ECLS) can be done in less than 15 min during ongoing cardiopulmonary resuscitation (CPR) [27]. The main advantage of this approach is to stabilise the patient until ROSC and until the damaged organ systems are able to regenerate. Furthermore, eCPR may help to gain time for diagnostic and therapeutic procedures that can address the underlying cause of the cardiac arrest. Observational studies suggest that in selected OHCA patients, eCPR might be associated with an increase in survival rates of up to 30% [28–30]. A meta-analysis showed a 13% increase in survival of eCPR compared to conventional CPR [31]. Until now, two randomised controlled trials (RCTs) investigating the clinical effects of eCPR are published: in the single centre, randomised ARREST trial including 30 patients with OHCA and refractory ventricular fibrillation, early eCPR significantly improved survival to hospital discharge compared with standard advanced cardiac life support treatment [32]. The Prague-OHCA trial [NCT01511666] compared an invasive approach including prehospital intraarrest hypothermia, mechanical chest compression device, ECLS and early invasive investigation and treatment with a standard approach. The results showed a trend towards an improved primary endpoint (survival with a normal or a favourable neurological outcome) for the invasive approach. The secondary endpoint, survival with good neurological outcome after 30 days, was observed significantly more often in the hyperinvasive group [33]. Current guidelines give no general recommendation for the use of eCPR [9, 27, 34, 35] and emphasise that the far-reaching decision whether or not to establish eCPR should always be taken by a multidisciplinary team considering all the available indicators. Another important point is that until now, no validated criteria for patient selection are available. Identification of predictors for a clinical benefit remains an important challenge. A meta-analysis investigated different prognostic factors, which might be helpful for the decision whether or not to start eCPR [36]. With G-CAR, we aim to identify possible predictors of clinical improvement, which will help to identify patients who benefit from eCPR. Other unanswered questions are whether in selected patients, an accelerated CPR algorithm with a focus on a fast transport (“load and go” principle) might be associated with an improved survival or whether an ECLS-Team should be active already pre-clinically. These questions are going to be addressed in ongoing and future clinical trials (e.g. INCEPTION-trial [NCT03101787], EROCA-trial [NCT03065647], ACPAR2-trial [NCT02527031]).

### Post-hospital phase

Psychosocial aspects such as health-related quality of life and the presence of psychopathological symptoms are important sequelae of intensive care treatment [15]. These symptoms and the predictors for their presence are not covered by existing registries. G-CAR aims to investigate these aspects by postal dispatch of standardised questionnaires and by telephone interviews after 6 and 12 months. Health-related quality of life will be investigated with the EQ-5D. Affective disorders will be analysed with the Hospital Anxiety and Depression Scale (HADS); post-traumatic stress syndrome with the Post-Traumatic Stress Syndrome 14-Questions Inventory (PTSS-14) and cognitive impairment will be investigated with the Montreal Cognitive Assessment (T-MoCA). Social reintegration will be assessed by Reintegration to Normal Living Index (RNLI).

## Summary

G-CAR is a multicentre registry for adult OHCA patients. It will record the prehospital and hospital course including aspects on eCPR. Furthermore, the post-hospital phase with a focus on several psychosocial aspects will be addressed. In parallel to the pilot phase of 24 months, a national extension of G-CAR is envisaged. Primary aim of G-CAR is a better understanding of the disease entity OHCA with the perspective of optimised outcomes.
